# A composite robotic-based measure of upper limb proprioception

**DOI:** 10.1186/s12984-017-0329-8

**Published:** 2017-11-13

**Authors:** Jeffrey M. Kenzie, Jennifer A. Semrau, Michael D. Hill, Stephen H. Scott, Sean P. Dukelow

**Affiliations:** 1 Department of Clinical Neurosciences, Hotchkiss Brain Institute, Cumming School of Medicine, Faculty of Medicine, University of Calgary, Calgary, AB Canada; 2 Calgary Stroke Program, Departments of Clinical Neurosciences, Radiology, Community Health Sciences, Hotchkiss Brain Institute, University of Calgary, Calgary, AB Canada; 3 Department of Biomedical and Molecular Sciences, Queen’s University, Kingston, ON Canada

**Keywords:** Proprioception, Stroke, Kinesthesia, Outcome measure, Position sense, Upper extremity, Robotics

## Abstract

**Background:**

Proprioception is the sense of the position and movement of our limbs, and is vital for executing coordinated movements. Proprioceptive disorders are common following stroke, but clinical tests for measuring impairments in proprioception are simple ordinal scales that are unreliable and relatively crude. We developed and validated specific kinematic parameters to quantify proprioception and compared two common metrics, Euclidean and Mahalanobis distances, to combine these parameters into an overall summary score of proprioception.

**Methods:**

We used the KINARM robotic exoskeleton to assess proprioception of the upper limb in subjects with stroke (*N* = 285. Mean days post-stroke = 12 ± 15). Two aspects of proprioception (position sense and kinesthetic sense) were tested using two mirror-matching tasks without vision. The tasks produced 12 parameters to quantify position sense and eight to quantify kinesthesia. The Euclidean and Mahalanobis distances of the z-scores for these parameters were computed each for position sense, kinesthetic sense, and overall proprioceptive function (average score of position and kinesthetic sense).

**Results:**

A high proportion of stroke subjects were impaired on position matching (57%), kinesthetic matching (65%), and overall proprioception (62%). Robotic tasks were significantly correlated with clinical measures of upper extremity proprioception, motor impairment, and overall functional independence. Composite scores derived from the Euclidean distance and Mahalanobis distance showed strong content validity as they were highly correlated (*r* = 0.97–0.99).

**Conclusions:**

We have outlined a composite measure of upper extremity proprioception to provide a single continuous outcome measure of proprioceptive function for use in clinical trials of rehabilitation. Multiple aspects of proprioception including sense of position, direction, speed, and amplitude of movement were incorporated into this measure. Despite similarities in the scores obtained with these two distance metrics, the Mahalanobis distance was preferred.

## Background

Stroke is heterogeneous, affecting sensory, motor, and cognitive functions that are required for daily activities. While there are well validated tools to assess motor and speech functions (eg. Fugl-Meyer Assessment (FMA) [1], the National Institute of Health Stroke Scale (NIHSS) [2], Chedoke-McMaster Stroke Assessment Impairment Inventory (CMSA) [3]) the use of high quality, validated assessment tools for measuring sensory function post-stroke (proprioception in particular) is limited [4], and there is still a lack of a gold standard assessment. While the FMA and NIHSS have sensory components to the assessment, they are seldom used as a sole measure of sensory impairment in research studies focused on sensation as they are based on relatively coarse scales. Yet, sensory and proprioceptive impairments have a significant negative impact on functional recovery following stroke [5–9]. Individuals with sensory and motor impairments, compared to those with just motor impairments, have longer lengths of hospitalization and fewer discharges home [10–12]. Furthermore, it has recently been shown that motor and proprioceptive impairments can occur independently after stroke [13].

Some commonly used clinical assessments of proprioception post-stroke include: 1) simple passive limb movement detection test [14] in which an examiner moves a subject’s limb segment with their eyes closed, and subjects are asked to say which direction the limb was moved; 2) the Revised Nottingham Sensory Assessment [15, 16] in which the subject is asked to mirror match the movement of a passively moved limb by a therapist; and 3) the Thumb Localizing Test [17] which involves passive movement of a subject’s arm and hand to a random position overhead, and is followed by subjects reaching to grasp their thumb with the opposite (less affected) hand. These assessments are scored crudely as normal, slightly impaired, or absent, and lack the sensitivity to detect smaller changes in proprioceptive function in part due to poor inter- and intrarater reliability [18, 19]. Therefore, establishing an objective and reproducible method to assess proprioceptive impairments post-stroke is vital to evaluating the efficacy of different treatments.

Other more advanced methods to assess proprioception have been developed [20–23], with many using robotic technology to measure the kinematics of an individual’s movements. Assessment devices can now measure position sense and kinesthetic impairments after stroke using arm contralateral matching [13, 24–26], in which a subject’s affected arm is passively moved by the robot to a position, and the subject mirror-matches the movement/position with their less affected limb. Another paradigm involves passive movement of a subject’s limb to a specified position, returning the limb to the starting position, and then having subjects actively move the same arm to this remembered position [21, 26]. This method has an advantage in that it does not require interhemispheric transfer of information, but has limited value in assessing people with concurrent motor deficits, or in assessing kinematic aspects of proprioception, such movement speed and amplitude perception. Further, results can be confounded by problems with spatial working memory. Threshold for detection of passive movement paradigms have also been used to assess proprioception [27, 28]. This paradigm eliminates confounds due to motor impairment and interhemispheric transfer of information but again, little information about the kinematics of movement perception (e.g. speed or direction) are gained from this task, and it typically takes much longer to complete than position/movement matching. Lastly, Carey et al. [20] have developed and validated a wrist position sense test, where a subject’s wrist is moved to a position (wrist flexion or extension) and without vision of the wrist the subject has to use their other arm to move a cursor to the direction the wrist is pointing. This method minimizes confounds due to interhemispheric information transfer and motor deficits, but again does not provide information about kinesthetic impairments.

Many of these assessments are reliable, reproducible, objective, and provide quantitative measures of proprioceptive function in the upper limbs. Dukelow et al. [13, 24], used a KINARM robot (BKIN Technologies, Kingston, ON), and detailed a contralateral position-matching task for the upper extremities that can measure various aspects of an individual’s position sense including: absolute error, variability in matching positions, systematic shifts in perceived workspace, and perceived contraction or expansion of the workspace. Similarly, Semrau et al. [25] recently detailed a kinesthetic matching task using the KINARM robot that can measure an individual’s ability to mirror-match the speed, direction, and amplitude of a robotically moved limb [8, 25]. These tasks are reliable [24], and provide numerous parameters that describe an individual’s position or kinesthetic sense impairments and can be used to guide a rehabilitation program tailored to the individual. Furthermore, these studies have shown a strong relationship between proprioceptive impairments and functional independence post-stroke, yet proprioceptive impairments are often not addressed in day-to-day therapy. Reliable and quantitative assessment tools are therefore critical for testing the efficacy of rehabilitation treatments, as in clinical rehabilitation trials.

While multiple kinematic parameters can provide a level of exactness around the nature of an individual’s proprioceptive impairments and are helpful for rehabilitation planning, a summary measure is needed for clinical therapeutic trials in rehabilitation. Thus, a single continuous metric of upper limb proprioceptive function that combines all parameters from the position and kinesthetic matching robotic tasks was developed using two common measures of distance, Euclidean distance (EDist) and Mahalanobis distance (MDist) [29]. The EDist was chosen as it is an easily interpretable calculation and considers each parameter independently. It is the square root of the sum of squared distances between data points (i.e. the straight-line distance between two points in three-dimensional space). The MDist is the next measure we used to compare with the EDist. It was chosen because the calculation accounts for correlations between parameters (by using the inverse of the variance-covariance matrix of the data set of interest), therefore preventing the overweighting of correlated parameters in the calculation. It is the distance between a point and the center of a distribution, measured along the major axes of variation (i.e. the standard deviation of an object in more than one dimension) [30, 31].. Because the kinematic parameters derived from the robotic tasks may demonstrate some degree of correlation with one another [13], the MDist can account for this auto-correlation. Theoretically, it should perform better at identifying stroke subjects who perform abnormally on the tasks and those who have atypical patterns of behavior relative to controls. The MDist is generally preferred over the EDist for multivariable data since it can cope with different structures of data [31].

MDist (or variants of it) has recently been used in other studies when examining reaching movements after stroke [32].. Our primary aim was to examine differences and similarities between two summary scores (EDist and MDist) in their ability to differentiate proprioceptive impairment in individuals with stroke from controls in a large patient sample. We hypothesized that using a composite proprioception score calculated from the Mahalanobis distance would more accurately identify impaired proprioception in individuals with stroke compared to a proprioception score calculated from the Euclidean distance.

## Methods

### Subjects

Subjects with stroke were recruited from the Foothills Medical Centre or Dr. Vernon Fanning Centre in Calgary AB, Canada. Inclusion criteria were: Subjects 18 years and older with first reported ischemic or hemorrhagic stroke. Exclusion criteria were: stroke affecting both hemispheres of the brain, upper limb orthopedic injury, neuropathy, evidence of apraxia [33], any other neurological disease or injury (e.g. Parkinson’s Disease, Multiple Sclerosis), unable to follow task instructions due to aphasia or cognitive impairments or significant fatigue which limited task performance. A sample of healthy control subjects without history of neurological injury or disease were also recruited from the community. Subjects provided written informed consent prior to study participation and this research was approved by The University of Calgary Conjoint Health Research Ethics Board (CHREB: #22123).

### Robotic assessments

**Fig. 1:**
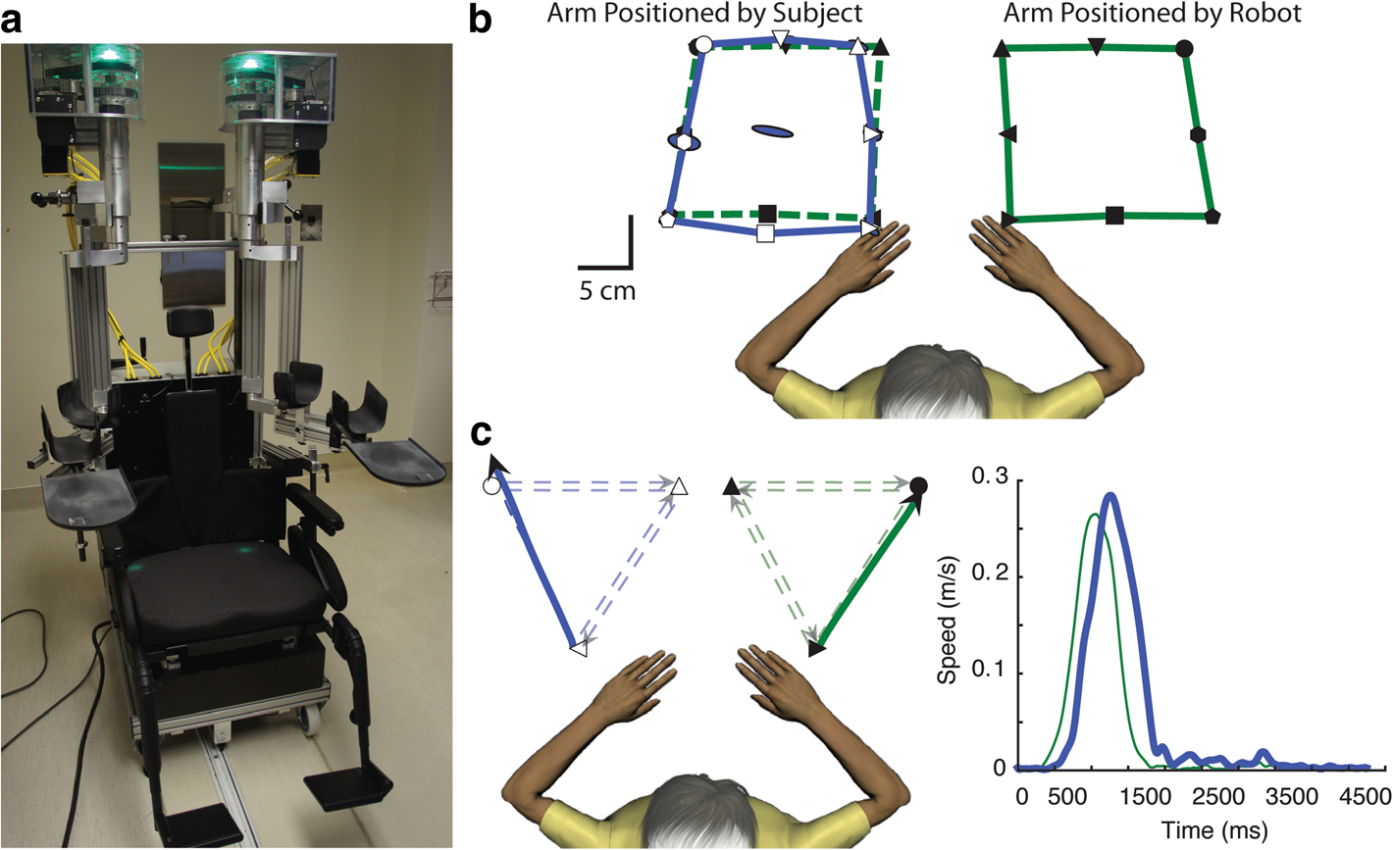
**a** KINARM robotic exoskeleton (BKIN Technologies, Kingston, ON, Canda). Subjects are seated in the wheelchair base with arms supported by the arm troughs. **b** Top-down view of the position matching task. The stroke affected arm was positioned by the robot (black targets, green lines) and subjects were required to mirror-match the target positions with their opposite hand (open targets, blue lines). Nine targets were matched to six times each for a total of 54 trials, presented in pseudorandom order. **c** Top-down view of an exemplar subject performing one trial of the kinesthetic matching task. The stroke affected arm was moved by the robot between two targets (green lines) and subjects were required to mirror match the speed, direction, and amplitude of movement as soon as they felt the robot move their arm (blue lines). The speed versus time profile represents the temporal aspects of the task, by measuring the response latency (time to initiation of the active arm movement) and peak speed ratio (difference between peak speeds of the passive (green) and active (blue) hands)

Assessment of proprioception was performed using a KINARM robotic exoskeleton (BKIN, Kingston, ON, Canada) (Fig. 1). Subjects were seated in the wheelchair base with both arms supported against gravity by arm troughs. The device was fitted to each subject by a trained study physician or therapist. Subjects were then wheeled into a virtual reality environment where vision of the upper extremities was occluded with a screen and bib fitted around the subject’s neck. The set-up of each subject and calibration of the robot took between six and eight minutes to complete. The position matching task took on average three minutes to complete and the kinesthetic matching task on average took five minutes to complete.

#### Arm position matching

The position matching task required subjects to mirror-match the *position* of a robotically moved arm (passive arm) with their opposite arm (active arm) [13, 24, 34]. The robot passively moved a subject’s stroke-affected arm to one of nine pre-determined positions in the workspace in a pseudorandom order (Fig. 1b). Subjects were then instructed to mirror-match the position of the passive arm with the opposite limb, without using vision. Six trials were performed for each of the nine target locations for a total of 54 trials.

The following parameters were used to quantify task performance after completion of all trials. *Absolute error*: the mean absolute distance error in the X (Abs_X_), Y (Abs_Y_), and XY directions (Abs_XY_) across all trials between the active arm and the ideal target position:

$$ {Abs}_{XY}=\sqrt{Abs_X^2+{Abs}_Y^2} $$

*Variability*: the trial-to-trial variability in matching to the same target position. Variability was calculated as the standard deviation of the active hand for each target position, and then averaged across all target positions for the X (Var_X_), Y (Var_Y_), and XY combined (Var_XY_) directions:

$$ {Var}_{XY}=\sqrt{Var_X^2+{Var}_Y^2} $$

*Contraction/ Expansion Ratio*: a measure of whether a subject perceived contraction or expansion of the workspace. It was calculated as the matched area of the active arm, relative to the area of the passive arm (Fig. 1b). Contraction/ Expansion Ratio was also calculated in the X (Contr/exp_x_), Y (Contr/exp_Y_), and combined XY (Contr/exp_XY_) directions:

$$ Contr/{\mathit{\exp}}_X=\frac{range_{x\_ active}}{range_{x\_ passive}} $$

*Systematic Shift*: the mean perceived translation of the workspace. The mean error between passive and active hands was calculated for each target position, followed by taking the mean of means across target locations. These were computed for the X (Shift_X_), Y (Shift_y_), and XY (Shift_XY_) directions:

$$ {Shift}_{XY}=\sqrt{Shift_x^2+{Shift}_y^2} $$

Each of these four parameters, taken in three directions (X, Y, and XY), provided a total of 12 parameters for the position matching task.

#### Arm kinesthetic matching

Kinesthetic matching measured a subject’s ability to mirror-match the *movement* of a robotically moved arm (passive arm) with the opposite arm (active arm), without using vision (Fig. 1c,d). This task has been previously described [8, 25, 35]. The passive arm was always the stroke-affected arm in our sample. Prior to the start of each trial, both arms were positioned at mirrored starting positions in the workspace. During this ‘positioning’ phase, the robot moved the passive arm to one of three positions in the workspace. Then two circular dots were illuminated on the projection screen, a white dot representing the active arm’s index finger, and a red dot representing the mirrored starting position. Subjects were instructed to place the white dot in the red dot. The targets were then extinguished, and after a random delay (1500 ± 25 ms) the passive arm was moved with a bell-shaped velocity profile (peak speed = 0.28 m/s) (Fig. 1d) between two pre-set target locations (20 cm) (Fig. 1d). Subjects were instructed to mirror-match the speed, direction, and amplitude of the passive arm with their active arm as soon as they felt the robot move their arm. Six movement directions were tested in a pseudorandom format to each of the three targets, with each direction being tested six times for a total of 36 trials. Kinematic data was filtered using 6th double-pass Butterworth filter with an overall 3 dB cutoff frequency of 10 Hz.

We quantified active arm movement (mirror matching) using the following parameters. *Response Latency (RL)*: the time between the onset of passive and active arm movements. *Initial Direction Error (IDE):* the angular deviation from subjects’ hand path at peak hand speed compared to the ideal hand movement path. *Peak Speed Ratio (PSR)*: the ratio of the maximum speed of the passive arm to the active arm. Ratios greater than one indicated a maximum speed of the active arm that was greater than the passive arm. *Path Length Ratio (PLR)*: the ratio of the distance travelled by the active arm relative to the distance travelled by the passive arm. Ratios greater than one indicated an active arm movement longer than the passive arm. The mean and standard deviation (variability) across the 36 trials for each of these four parameters were calculated as separate parameters (e.g. RL: mean response latency, RLv: response latency variability). Thus, a total of eight parameters were derived from the kinesthetic matching task [25].

### Development of composite score

The parameters chosen to be included in the composite score were based on early observations of patterns of behavior that individuals showed post-stroke. These parameters have been reported in previous studies and we wanted to be consistent with our previous work [8, 13, 24, 25, 35–37].

#### Conversion of parameter scores in native units to z-scores

Scoring systems were developed that captured subject performance relative to that observed for neurologically intact subjects. The first step was to convert the task parameters from their native units to normalized z-scores based on a large sample of neurologically intact control subjects (*n* = 160, tested on both arms = 319 data points, median age = 54 (range = 18–93), female = 84, right handed = 147, left handed = 13). Performance metrics for this sample of healthy subjects were transformed to a normal distribution, using a Box-Cox power transformation [38–40]. Linear regressions were then used to consider the influence of age for each parameter, and then verified that the data was normally distributed. If necessary, the Box-Cox transformations were adjusted to achieve normality. Data points ± 3.29 standard deviations from the mean were considered outliers and were removed from the control dataset (maximum <4% of subjects per parameter, average < 1% per parameter). This entire process was performed again after any outlier removal.

The next stage involved transforming these z-scores so that a score of 0 was equal to the best possible performance and higher scores indicated worse performance. This is because some of the task parameters were one-sided in which negative z-scores indicated better performance (e.g. initial direction error for kinesthetic matching), whereas others were two-sided in which both positive and negative z-scores of increasing value indicated worse performance (e.g. contraction/expansion ratio for position matching). Therefore, z-scores for the one-sided parameters (e.g. Position matching: Abs, Var, Shift. Kinesthetic matching: RL and IDE), were transformed such that negative infinity was equal to zero and positive infinity remained the same (henceforth referred to as zeta-scores). The zeta-scores for the two-sided parameters were simply equal to the z-scores. These zeta-scores were used in the subsequent composite score calculations. For the arm position matching task, these values were computed using automated analysis tools from KINARM Standard Tests (BKIN Technologies). For the kinesthetic task, values were computed in MATLAB (v2014b, MathWorks, Natick, MA) using custom routines from BKIN Technologies.

#### Composite score 1: E-score based on Euclidean distance

The Euclidean Distance (EDist) was computed from the healthy control subjects for a given task.. This EDist is simply the root mean square (RMS) of the zeta-scores for all parameters associated with a task:

$$ EDist=\sqrt{(a)^2+{(b)}^2+\dots } $$

where a, b, etc. represent the zeta-scores for a subject. EDist increases in size with the number of parameters. In order to compare scores across tasks, the Box-Cox equations were used to convert the EDist scores for the healthy control population into a normal distribution (followed by testing for normality). These scores were again transformed to all positive values and scores ≥3.29 were considered outliers and were removed. This process was repeated until no outliers remained in the distribution (~1% of subjects removed). Similar to the zeta-scores, a final E-Score of 0 signifies best performance and increasing positive values signifies poorer performance. The units follow the same percentiles as ±1SD of a normal distribution (i.e. 1 = 68.3%, 2 = 95.4%, etc.).

#### Composite score 2: M-score based on Mahalanobis distance

The Mahalanobis Distance is similar to the Euclidean Distance measure above, except that the covariance matrix was used to consider correlations between parameters [29]. As in the E-Score processing, the z scores were first transformed into positive values with 0 reflecting best performance and increasing values reflecting poorer performance (zeta scores). This MDist is computed using the zeta-scores of all parameters from a task using:

$$ MDist=\sqrt{(x){C}_x^{-1}{(x)}^T} $$

Where *x* is the row vector of zeta scores for an individual subject, and *C*_*x*_ is the covariance matrix computed from the healthy control population dataset [30]. MDist values were converted into an M-Score following the same procedures used to convert EDist to E-Score.

E- and M-Scores were generated for position matching and kinesthetic matching separately. Subjects were considered ‘impaired’ on the task if they received a score greater than 1.96, indicating their performance was more than 95% from the mean of neurologically intact control subjects. The overall proprioception score was the average between the position and kinesthetic matching scores. All statistical analyses and calculations were performed in MATLAB (v2014b, MathWorks, Natick, MA) using both custom scripts and scripts from BKIN Technologies. The BKIN Dexterit-E User Guide refers to the E-Score as the ‘Task Score’ while the M-Score is the M-Score.

### Clinical Assessments

A battery of clinical assessments was performed on subjects with stroke by a trained study physician or therapist. The Chedoke McMaster Stroke Assessment (CMSA) for the Upper Extremities was performed to evaluate upper limb motor function [3]. The Functional Independence Measure (FIM) was used as a metric for independence within activities of daily living [41]. The conventional subtests of the Behavioral Inattention Test (BIT) was used to evaluate visuospatial neglect [42]. We included this clinical assessment of visuospatial neglect because we have previously noted that there can be a high co-occurrence of visuospatial neglect and sensory loss [35]. Handedness was measured using the Modified Edinburg Handedness Inventory (performed on healthy controls as well) [43]. Lastly, the Thumb Localizing Test (TLT) was used to evaluate upper limb proprioceptive function [17]. For this test, the subject’s eyes were closed and the subject’s stroke-affected limb was placed somewhere in space above eye level by a therapist. Subjects were then instructed to grasp this thumb with their opposite (i.e. less affected) hand. Performance was scored on an ordinal scale from zero (no difficulty locating thumb) to three (unable to locate thumb). We choose this assessment because it was easy to administer and uses both limbs to test proprioception, akin to our robotic tasks. There is currently no gold standard for the assessment of upper limb proprioception post-stroke. The level of agreement between the TLT and robotic assessments in classifying subjects as having impaired proprioception (TLT > 0, robotic score > 1.96) was calculated using Cohen’s Kappa [44]. Comparisons between robotic and clinical measures were performed using Pearson or Spearman correlations, where appropriate, with Bonferroni corrections for multiple comparisons. The strength of association was classified as either very weak (*r* = 0.00–0.19), weak (*r* = 0.20–0.39), moderate (*r* = 0.40–0.59), strong (*r* = 0.60–0.79), or very strong (*r* = 0.80–1.0) [45].

## Results

### Subjects

A total of 285 stroke subjects (Female = 92) were recruited and assessed on the position matching and kinesthetic matching tasks (Table 1).

**Table 1:** Demographic and clinical information for sample of 285 subjects with stroke. Values are presented as mean ± standard deviation, or a count of the number of subjects in each category

	Left Hemisphere Stroke (*n* = 115)	Right Hemisphere Stroke (*n* = 170)	Total (*n* = 285)
Age	59.5 ± 14.7	61.2 ± 14.6	60.6 ± 14.6
Sex (F, M)	41, 74	51, 119	92, 193
Handedness (R, L, Mixed)	104, 10, 1	160, 8, 2	264, 18, 3
Days post-stroke	12 ± 18	12 ± 12	12 ± 15
CMSA^a^ (1,2,3,4,5,6,7)	10,6,15,7,20,15,40	14,19,18,6,37,24,51	24,25,33,13,57,39,91
FIM	115.1 ± 17.5	112.1 ± 18.5	113.3 ± 19.6
TLT (0,1,2,3)	60,31,17,5	82,52,25,11	142,83,42,16
BIT	138.1 ± 16.3	130.1 ± 21	133.3 ± 19.6
Arterial Territory (MCA, PCA, ACA, VA)^b^	73,18,7,21	124,28,4, 24	197,46,11,45

*F:* Female, *M:* Male, *R:* Right, *L:* Left, *CMSA:* Chedoke McMaster Stroke Assessment for the Upper Extremities, *FIM:* Functional Independence Measure, *TLT:* Thumb Localizing Test, *BIT:* Behavioral Inattention Test, *MCA:* Middle Cerebral Artery, *PCA:* Posterior Cerebral Artery, *ACA:* Anterior Cerebral Artery, *VA:* Vertebral Artery

^a^Values are for the stroke-affected limb

^b^Vertebral artery territory includes any artery supplied by the vertebral artery, before branching into the posterior cerebral arteries (i.e. posterior inferior cerebellar artery, anterior inferior cerebellar artery, basilar artery). Thirteen subjects were classified as having strokes in more than one arterial territory

### E- and M-scores

**Fig. 2:**
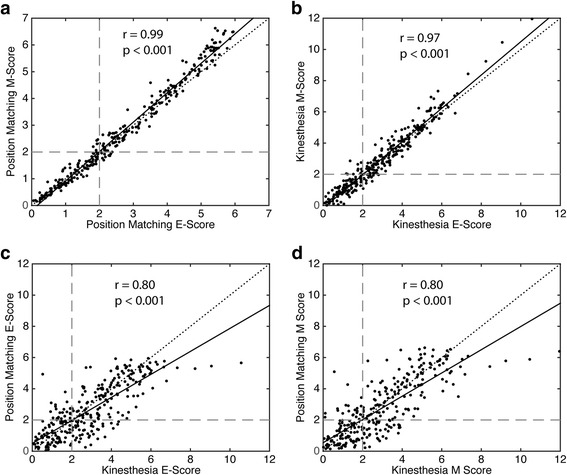
Scatter plots of robotics scores for individual stroke subjects (*N* = 285). Greater scores indicate worse proprioception **a** The relationship between position matching performance calculated using subjects’ E-Scores (Euclidean distance of an individual subject’s robotic scores from the mean healthy control scores) versus M-Scores (Mahalanobis distance of an individual subject’s robotic scores from the mean healthy control scores). **b** Relationship between kinesthetic matching performance calculated using the E-Scores and M-Scores. **c** Relationship between the position matching and kinesthetic matching tasks based on the E-Scores. **d** Relationship between the position matching and kinesthetic matching tasks based on the M-Scores. E and M-Scores represent standard deviations from the mean of neurologically intact control performance. Grey dashed lines indicate 1.96 standard deviations. Data points beyond 1.96 indicate impaired performance. Black dotted lines on each plot indicate unity between scores, black solid lines on each plot indicate least squares fit between scores. Pearson correlation coefficients (r) and associated *p*-values (p) are presented in each plot

The composite E- and M-Scores were highly correlated with one another (Fig. 2a and b). There were strong positive, linear relationships between E- and M-Scores on the position matching (*r* = 0.99, *p* < 0.001) and kinesthetic matching (*r* = 0.97, *p* < 0.001) tasks. E- and M-Scores on the position matching and kinesthesia tasks were also positively correlated with one another (*r* = 0.80, *p* < 0.001). Despite these high correlations, there were instances in the overall proprioception score where subjects were considered ‘impaired’ (score > 1.96) based on the E-Score and not the M-Score (*n* = 11), and vice versa (*n* = 6). However, these discrepancies were rarely larger than ± one standard deviation.

Comparing performance between tasks revealed that performance on position matching was not always indicative of performance on kinesthetic matching (Fig. 2c and d). More subjects had impaired kinesthetic matching with unimpaired position matching (*n* = 45 using E-Score; *n* = 44 using M-Score) than subjects who demonstrated impaired position matching with unimpaired kinesthetic matching (*n* = 24 using E-Score; *n* = 23 using M-Score). More subjects with right hemisphere stroke were abnormal on the position matching task (E-Score = 73.4%, M-Score = 69.8%), kinesthetic matching task (E-Score = 74.6%, M-Score = 74.0%), and overall (E-Score = 75.1%, M-Score = 73.4%), relative to subjects with left hemisphere stroke (Position match: E-Score = 35.3%, M-Score = 38.8%; Kinesthesia: E-Score = 51.7%, M-Score = 50.9%; Overall: E-Score = 47.4%, M-Score = 45.7%). Overall, more subjects were abnormal on the kinesthetic matching task (E-Score = 65.3%, M-Score = 64.6%) relative to the position matching task (E-Score = 57.9%, M-Score = 57.2%).

Table 2 shows Pearson correlations between z-scores for each of the position and kinesthetic matching parameters. The E- and M-Scores for position and kinesthetic matching were positively correlated with one another (E-Scores, *r* = 0.80, *p* < 0.001. M-Scores, *r* = 0.80, *p* < 0.001). The overall proprioception score was calculated as the average of the position matching and kinesthetic matching scores. The average E-Scores identified 63.9% of subjects as abnormal (score > 1.96) while the average M-Scores identified 62.1% of subjects as abnormal.

**Table 2:** Pearson correlation coefficients between position matching and kinesthetic matching parameters for subjects with stroke (*n* = 285). Comparisons were made between z-scores for each task parameter. Z-scores were calculated based on distributions of neurologically intact control subject scores (*n* = 319 data points)

		Position Matching Parameters													
		Absolute Error			Variability			Contr/Exp			Shift			E	M
Kinesthetic Matching Parameters		X	Y	XY	X	Y	XY	X	Y	XY	X	Y	XY		
	IDE	**0.65**	**0.65**	**0.69**	**0.65**	**0.63**	**0.67**	**0.58**	**0.57**	**0.60**	**0.24**	**0.38**	**0.38**	**0.80**	**0.80**
	IDEv	**0.59**	**0.50**	**0.60**	**0.57**	**0.56**	**0.59**	**0.49**	**0.45**	**0.49**	**0.22**	**0.22**	**0.31**	**0.67**	**0.66**
	PLR	**0.34**	**0.40**	**0.39**	**0.40**	**0.35**	**0.40**	**0.31**	**0.44**	**0.36**	0.09	**0.29**	0.20	**0.47**	**0.48**
	PLRv	**0.56**	**0.55**	**0.61**	**0.60**	**0.58**	**0.61**	**0.40**	**0.46**	**0.41**	**0.26**	**0.29**	**0.36**	**0.65**	**0.64**
	RL	**0.43**	**0.43**	**0.45**	**0.46**	**0.46**	**0.47**	**0.37**	**0.32**	**0.38**	0.10	0.18	0.18	**0.50**	**0.49**
	RLv	0.12	0.11	0.12	0.13	0.18	0.14	0.15	0.14	0.14	−0.06	−0.09	−0.02	0.18	0.17
	PSR	**0.25**	**0.36**	**0.30**	**0.22**	**0.27**	**0.24**	**0.39**	**0.39**	**0.40**	0.12	**0.24**	0.19	**0.44**	**0.47**
	PSRv	0.17	0.09	0.16	**0.23**	0.21	**0.23**	−0.05	−0.03	−0.03	0.11	0.05	0.15	0.08	0.05
	E	**0.63**	**0.66**	**0.69**	**0.66**	**0.64**	**0.68**	**0.57**	**0.60**	**0.60**	**0.23**	**0.38**	**0.37**	**0.80**	**0.80**
	M	**0.61**	**0.66**	**0.66**	**0.64**	**0.63**	**0.66**	**0.57**	**0.60**	**0.59**	**0.21**	**0.40**	**0.36**	**0.79**	**0.80**

All bold values are significant at *p* < 0.00036 (Bonferonni corrected, *p* < 0.05, *n* = 140 comparisons)

*IDE(v):* Initial Direction Error (variability), *PLR(v):* Path Length Ratio (variability), *RL(v):* Response Latency (variability), *PSR(v):* Peak Speed Ratio (variability). *Contr/Exp:* contraction/ expansion ratio. *E:* ‘E -score’ calculated from Euclidean distance of z-scores. *M:* ‘M -score’ calculated from Mahalanobis distance of z-scores

#### Comparison with clinical measures

The robotic proprioception measures showed moderate correlation with the clinical measures of proprioception, upper extremity arm function, and overall functional independence (Table 3). We also calculated the agreement between the thumb localizing test (TLT) and our robotic scores in classifying subjects as having ‘normal’ or ‘abnormal’ proprioception using Cohen’s Kappa [44]. Abnormal proprioception based on the TLT was any score greater than or equal to one, and abnormal proprioception on the robotic tasks was an E- or M-Score greater than 1.96. Table 3 shows the agreement between clinical and robotic classification of proprioceptive impairments.

**Table 3:** Spearman correlations between clinical and robotic assessment scores and the agreement between clinical and robotic classification of proprioceptive impairment in subjects with stroke. Values presented are Spearman’s rho for correlations and Cohen’s Kappa for level of agreement. Subjects were considered impaired on the robotic tasks if they scored >1.96, and impaired on the Thumb Localizing Task if they scored >0

	Robotic Assessments						
Clinical Assessments (ρ(283)=)		PM_E	PM_M	KIN_E	KIN_M	Overall E-Score	Overall M-Score
	TLT	0.48	0.49	0.47	0.48	0.50	0.51
	CMSA	−0.50	−0.50	−0.56	−0.58	−0.57	−0.57
	FIM	−0.40	−0.40	−0.44	−0.44	−0.44	−0.45
Agreement, (k=)	TLT	0.27	0.28	0.29	0.33	0.32	0.33

All values (correlations and agreement) are significant at *p* < 0.001

*TLT:* Thumb Localizing Task (scored from 0 = no impairment to 3 = unable to locate thumb). *CMSA:* Chedoke McMaster Stroke Assessment for the Upper Extremities (scored from 7 = normal movement to 1 = flaccid paralysis). *FIM:* Functional Independence Measure (scored from 126 = complete independence with daily activities to 18 = complete dependence/total assistance). PM_E: E-Score for the position matching task. PM_M: M-Score for the position matching task. KIN_E: E-Score for the kinesthesia task. KIN_M: M-Score for the kinesthesia task. Overall E- and M-Scores indicate the average score between the position matching and kinesthesia tasks

### Exemplar subjects

**Fig. 3:**
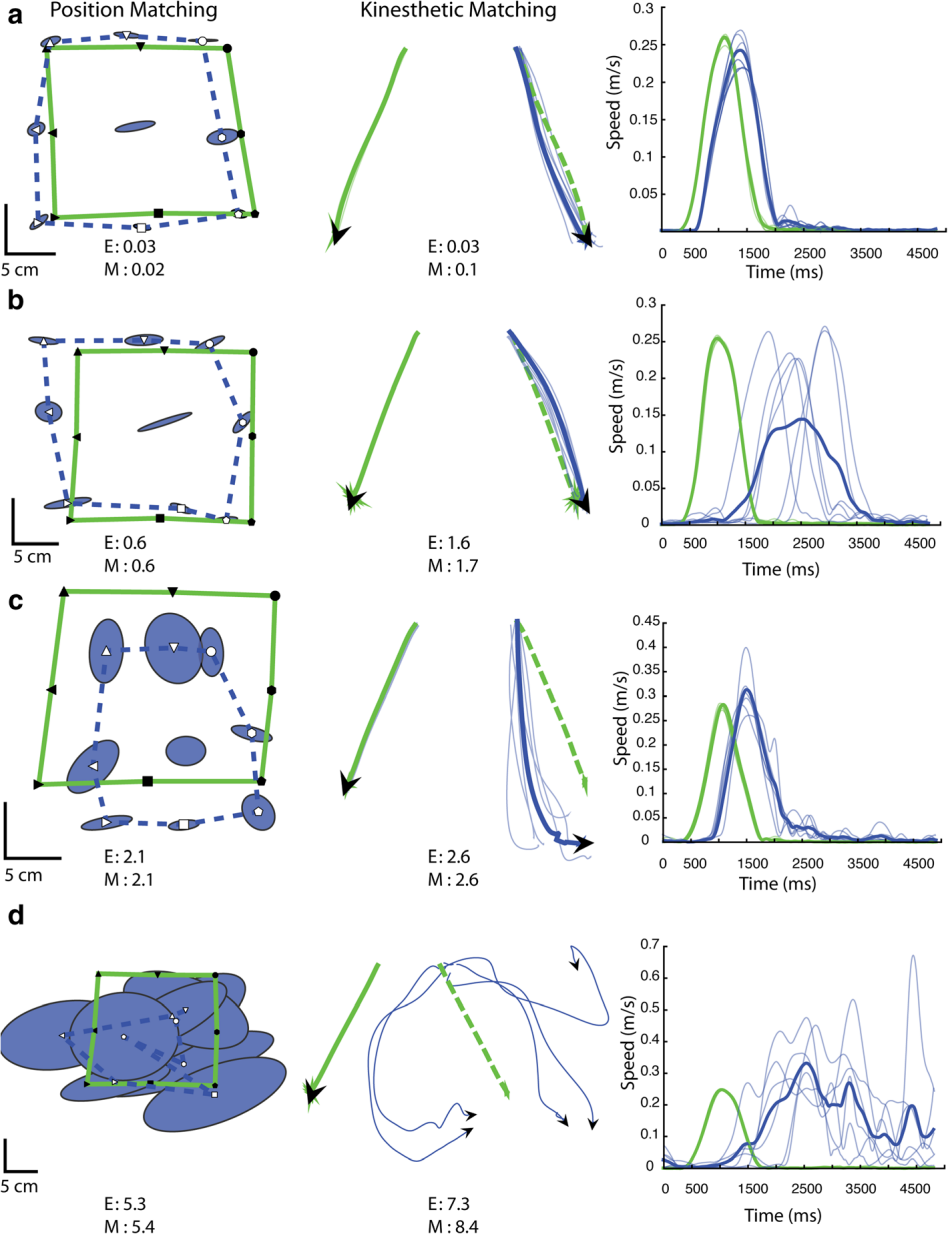
Exemplar subjects’ performance on the position (left panel) and kinesthetic (middle and right panel) matching tasks. For the position matching task, the subject’s matched hand positions (open targets, blue lines) are mirrored across the vertical centre line and displayed on top of the passive robotically moved hand positions (black filled targets, green lines). For the kinesthetic matching task, both hand movements are displayed where solid green lines indicate passive robotic movements, dotted green lines indicate the optimal movement path of the opposite arm, and solid blue lines indicate active subject movements. Light blue lines indicate individual trials and dark blue lines indicate the average between all completed trials in the given movement direction. Note that for the position matching task, the blue and green lines simply connect the target positions for display purposes and do not represent the hand movements between targets. E: ‘E-Score’ indicates the subject’s composite score calculated from the Euclidean distance. M: ‘M-Score’ indicates the subject’s composite score calculated from the Mahalanobis distance. **a** Control exemplar. Intact position matching performance is indicated by low variability (small ellipse size), with minimal shift or contraction/expansion of the workspace (blue dotted lines). Intact kinesthetic matching performance is indicated by alignment in movement direction to the ideal movement path, and a short response latency (onset of active arm movement) with similar peak speeds between passive (green lines) and active hands (blue lines). **b** Stroke subject with intact performance on the position matching task. This subject also performed well on the spatial aspects of kinesthesia (middle panel) but performed poorly on the temporal aspects of kinesthesia (right panel). **c** Stroke subject who performed poorly on the position matching task (increased variability and shift of workspace). This subject demonstrated impairments on the spatial aspects of kinesthesia but normal performance on the temporal parameters (short and consistent response latency and peak speeds). **d** Stroke subject who was severely impaired on both position and kinesthetic matching tasks

Figure 3 describes four individuals who performed differently on the position and kinesthetic matching tasks. Starting with the position matching task, a healthy control subject (Fig. 3a) mirror-matched the target positions accurately and consistently (denoted by the small ellipse sizes which indicate one standard deviation of error) (position matching E-Score = 0.03, M-Score = 0.02). A subject with stroke in Fig. 3b also demonstrated normal performance on the position matching task (E-Score = 0.6, M-Score = 0.6), while the subject with stroke in Fig. 3c demonstrated abnormal performance (E-Score = 2.1, M-Score = 2.1) resulting primarily from increased absolute error (Abs_Y_ z = 2.1), variability (Var_Y_ z = 3.4), and systematic shift (Shift_Y_ z = −2.4). Lastly, Fig. 3d presents a subject who was significantly impaired on the position matching task (E-Score = 5.3, M-Score = 5.4).

For the kinesthetic matching task (Fig. 3a), the control subject made smooth, straight movements in line with the ideal trajectory and demonstrated a consistent response latency and movement speed (E-Score = 0.03, M-Score = 0.1). The subject with stroke presented in Fig. 3b performed well on matching the direction (IDE z = −1.8) and amplitude (PLR z = 1.4) of passive movements, but poorly on response latency (RL z = 2.4) and response latency variability (RLv z = 2.3) (E-Score = 1.6, M-Score = 1.7). In comparison, the subject with stroke in Fig. 3d had difficulties in matching the direction (IDE z = 3.0) and length (PLRv z = 2.7) of passive movements, but performed well in matching speed (PSR z = −0.88) with normal and consistent response latency (RL z = 1.1, RLv z = 0.6) (E-Score = 2.6, M-Score = 2.6). Finally, the stroke subject in Fig. 3d was significantly impaired on all aspects of the kinesthetic matching task (E-Score = 7.3, M-Score = 8.4).

## Discussion

We have developed a composite measure of upper limb proprioception using the KINARM robotic exoskeleton that can be used as an outcome measure for tracking proprioceptive impairment over time and across subjects [24]. Despite the significant correlation between position sense and kinesthetic sense impairments, individuals after stroke were often impaired on different aspects of position sense and kinesthetic sense (Fig. 3), with some individuals demonstrating impairments in one sense and not the other (Fig. 2). Our robotic scores also identified more stroke subjects as having proprioceptive impairments (~62%) compared with standard clinical measures (50%). Contrary to our hypothesis, the Mahalanobis distance score identified slightly fewer subjects as impaired (62.1%) compared to the Euclidean distance score (63.9%).

There is currently no gold standard for assessing proprioceptive impairment after stroke, despite proprioceptive impairments being common (over 50%) [8, 10, 20, 24, 25] and having a strong relationship with functional recovery post-stroke [11, 12]. In order for clinical rehabilitation trials to identify appropriate treatments for improving proprioceptive function post-stroke, a sensitive and reliable outcome measure of proprioception is needed [19]. The level of agreement (k = 0.32–0.33, *p* < 0.001) between our robotic measures of proprioception and a commonly used clinical measure of proprioception (the Thumb Localizing Test) demonstrates some discrepancy between these tests in classifying subjects as normal or abnormal. Based on previous studies we expected fair agreement between these assessments [24, 25]. These results are not surprising given the known limitations with these clinical tests and their low reliability [18]. Unfortunately, there is currently no gold standard for assessing upper limb proprioception post-stroke. Our proprioception score, utilizing the KINARM and Mahalanobis distance, provides an overall indicator of proprioceptive impairment that considers multiple kinematic and spatial parameters. This score is suitable as a primary outcome measure of proprioception for use in clinical rehabilitation trials targeting upper limb function.

The Euclidean and Mahalanobis distances have been used for decades as general distance metrics, for outlier detection [46, 47], and in various classification algorithms [48–51]. The Mahalanobis distance was preferred over the Euclidean distance in summarizing our robotic parameters, because it takes into consideration correlations between parameters. Theoretically, it is more sensitive in identifying abnormal patterns of behavior compared to the Euclidean distance. It can also account for impaired performance that is in line with the normal variation in task performance, thus producing a lower overall score (i.e. more normal). This is likely why fewer subjects were identified as abnormal based on the M-Score compared with the E-Score, since impairments on parameters that were in line with normal variations in task performance had less of an impact on the overall score. Based on our data, EDist and MDist performed similarly in calculating a composite score from the robotic parameters, and neither method produced drastically inflated results compared to the other for any one subject. Recently, Kitago et al. (2015) used functional principal component analysis along with the Mahalanobis distance to create a single variable to measure reaching performance during a visually guided reaching paradigm in chronic stroke survivors. This type of data driven approach is useful for capturing kinematic aspects of movement (or impairments in movement) that may not be immediately apparent. However, we chose to use the Mahalanobis distance on previously defined kinematic parameters for two reasons. One was to maintain consistency with our previous work and that of others. The second reason was to ensure that the parameters used to construct the M-Score were behaviorally meaningful.

Some limitations exist with this study and with using composite scores. First, a composite score may not fully describe the nature of an individual’s impairment. Figure 3 shows that individuals post-stroke can be impaired on different aspects of proprioceptive sensation. There are also subjects who have difficulties with specific aspects of proprioception but are classified as normal based on the composite score. Thus, while a single task score might be necessary for planning and reporting clinical trials, it may not be informative enough when deciding on what an individual should be working on in a therapy intervention. Second, deciding on what the minimal clinically important difference is for the M-Score of proprioception requires further analysis comparing changes in M-Score with changes in an individual’s functional ability. Third, there is the possibility that fatigue may have contributed to the difference in performance between the position-matching and kinesthesia tasks, since the position-matching task was always assessed before the kinesthesia task in our protocol. However, we did not observe any decrease in performance over the course of the kinesthesia task across all subjects after visual inspection of the data. Additionally, given the position matching task takes only three minutes and the kinesthesia task takes five minutes, we suspect any fatigue in our subjects, if present at all, was minimal. Lastly, our composite score does not include the assessment of distal joints (e.g. wrist, thumb, and fingers). Assessment tools have been designed for proprioception at the distal joints (e.g. Wrist Position Sense Test) [20] but our focus here was on the shoulder and elbow joints. Proprioceptive impairments at the shoulder and elbow are related to functional independence [24, 25], however, future studies could examine the impact of better quantifying proprioception throughout the upper limb and the cumulative impact on prognosis and treatment planning.

Having tangible and easily interpreted outcome variables enables the translation of someone’s specific impairments directly to therapy, where a rehabilitation program can be tailored to these impairments. Somatosensory and proprioceptive impairments are becoming well known as significant factors in the recovery of function post-stroke. However, sensory retraining is still in its infancy with regards to high-quality clinical trials. There is a great need for improved outcome measures for proprioceptive impairments post-stroke and improved evidence for proprioceptive interventions [9, 19, 52, 53].

## Conclusions

We have developed a quantitative and reproducible outcome measure for upper limb proprioception that takes into consideration both position and kinesthetic senses. In a large sample of subjects with recent stroke (*n* = 285), over 60% had abnormal proprioception relative to a neurologically intact control population. The outcome measure presented here for proprioception will be important in measuring the efficacy of clinical stroke rehabilitation trials for improving proprioceptive function.

## Abbreviations

Abs: Absolute Error
BIT: Behavioral Inattention Test
CMSA: Chedoke McMaster Stroke Assessment
Contr/exp: Contraction Expansion Ratio
EDist: Euclidean distance
E-Score: Composite score calculated from the Euclidean Distance of z-scores
FIM: Functional Independence Measure
IDE(v): Initial Direction Error (variability)
MDist: Mahalanobis distance
M-Score: Composite score calculated from the Mahalanobis Distance of z-scores
PLR(v): Path Length Ratio (variability)
PSR(v): Peak Speed Ratio (variability)
RL(v): Response Latency (variability)
TLT: Thumb Localizing Test
Var: Variability
